# Multi-environment meta-analysis reveals the mechanism of action of potassium-solubilizing microorganisms on crop yields

**DOI:** 10.3389/fpls.2025.1659478

**Published:** 2025-11-03

**Authors:** Xing Fan, Yuanpeng Zhu, Yan Jia, Peishen Du, Weini Wang, Junmei Liu, Zhijun Lv, Ronghao Liu, Xiaobin Li

**Affiliations:** ^1^ College of Water Resources Science and Engineering, Taiyuan University of Technology, Taiyuan, China; ^2^ Shanxi Key Laboratory of Collaborative Utilization of River Basin Water Resources, Taiyuan, China; ^3^ Anhui Provincial Academy of Eco-Environmental Science Research, Hefei, China; ^4^ Ordos Agriculture and Animal Husbandry Ecology and Resource Protection Center, Ordos, Inner Mengulia, China; ^5^ Agricultural and Animal Husbandry Technology Extension Center, Dalat Banner, Ordos, Inner Mengulia, China; ^6^ Institute of Agricultural Resources and Regional Planning, Chinese Academy of Agricultural Sciences, Beijing, China

**Keywords:** potassium-solubilizing microorganisms, soil available potassium, crop yield, meta-analysis, sustainable agriculture

## Abstract

The availability of soil potassium plays a critical role in yield increases. Potassium-solubilizing microorganisms (KSM) offer a promising biological solution to improve potassium availability, but their efficacy across diverse global environments remains uncertain. Through a global meta-analysis of 102 studies (846 paired observations), we systematically evaluated the effects of KSM application on crop yields across five key dimensions: microorganism types, soil factors, crop classifications, field management, and stress types. KSM inoculation significantly increased soil available potassium (+28.9%), crop yield (+23.4%), and key growth indices, such as root length (+29.50%) and leaf area (+44.7%). This study identified *Aspergillus* spp. as the most suitable microorganism, and revealed that KSM efficacy is highly dependent on context: yield responses were greatest in clay loam soils, vegetable crops, and greenhouse conditions. Structural equation modeling indicated that microbial abundance, climate, soil available potassium, and plant growth (root length and leaf area) are key direct and indirect drivers of yield enhancement. The results indicate that the application of KSM is an effective strategy to increase crop yields in various environments. By identifying the optimal conditions for KSM application, the identification of optimal application parameters, derived from cross-study analysis, provides a robust strategy for leveraging microbial communities to boost soil potassium availability and nutrient efficiency, thereby contributing to the transition toward more sustainable and climate-resilient agriculture.

## 1 Introduction

Potassium (K) is an essential plant nutrient and the most abundant cation in plant cells, playing a critical role in a myriad of physiological and metabolic processes, including enzyme activation, osmotic regulation, and photosynthesis (Sheng, 2005; Sattar et al., 2019; Li et al., 2023b). Consequently, potassium availability directly governs photosynthetic efficiency, crop stress resistance, growth, and ultimately yield and quality (Yang et al., 2023; Wang et al., 2024; Zhao et al., 2024). However, the natural soil potassium pool often lacks sufficient capacity to convert insoluble K into plant-available forms, frequently failing to meet crop demand (Xu et al., 2025). This limitation represents a fundamental constraint on agricultural productivity and sustainability, contributing to inefficient fertilizer use and environmental imbalances (Zhao et al., 2024; Liu et al., 2025).

Addressing this challenge requires a shift from purely chemical solutions towards biological strategies that harness soil ecosystem processes. Potassium-solubilizing microorganisms (KSM) are a key functional group within the soil microbiome that can enhance mineral weathering and mobilize fixed potassium pools through biological mineralization (Sattar et al., 2019). Their mechanisms (including acid production, phytohormone secretion, and extracellular enzyme release) not only improve potassium availability but also enhance soil health and plant resilience (Meena et al., 2014; Sun et al., 2019; Chen et al., 2022). This positions KSM application at the intersection of microbial ecology and plant nutrition, offering a pathway to more sustainable agricultural intensification by working with, rather than against, soil biological processes (Shen et al., 2016).

Numerous targeted studies have demonstrated the efficacy of specific KSM strains (e.g., *Bacillus*, *Pseudomonas*) in increasing potassium solubility and crop yields under controlled or local conditions (Khanghahi et al., 2018; Sarikhani et al., 2018; Nawaz et al., 2023). However, the transition from promising microbe-level effects to reliable field-scale outcomes remains a major hurdle in microbial ecology applied to agriculture. A critical gap exists between demonstrating efficacy in discrete settings and predicting effectiveness across the heterogeneous landscapes of global agriculture. The intricate relationships between KSM functionality, environmental context (e.g., soil type, stress type), and field management are poorly quantified. Consequently, it is unclear whether the benefits of KSM are generalizable or contingent on specific, and often unknown, ecological interactions and agronomic conditions. This uncertainty hinders the development of predictive models and evidence-based recommendations for integrating KSM into sustainable farming systems.

To bridge this gap between microbial ecology and agricultural practice, we conducted a comprehensive global meta-analysis of studies from 2015-2024. By synthesizing evidence across five key dimensions: microbial species, soil types, crop types, field management, and stress types (**Table 1**). We aim to: (i) quantify the overarching impact of KSM on soil potassium dynamics and plant performance; (ii) identify the contextual factors that most strongly regulate KSM efficacy; and (iii) elucidate the complex pathways through which KSM influence crop yield, integrating microbial, plant, and environmental variables. Our work provides a mechanistic, evidence-based framework for understanding KSM ecology in agricultural systems, ultimately guiding the development of targeted microbial strategies for enhancing potassium use efficiency and advancing sustainable crop production.

**Table 1:** Subgroup classification table for the forest plots.

First-order factor	Secondary factor	Clusters				
		1	2	3	4	5
Microorganism	Potassium-solubilizing microorganisms	Bacillus	Enterobacteria	Aspergillus	Pseudomonas	Rhizobium
Soil factor	Soil texture	Sandy loam	Loam	Clay loam	–	–
	Soil pH	> 8	> 6–8	< 6	–	–
Crop classification	Crop category	Vegetable crop	Food crop	Fruit tree	–	–
	Crop family	Poaceae	Legume	Solanaceae	Cruciferous	Anacardiaceae
Field management	Planting method	Greenhouse experiment	Field experiment	–	–	–
Stress type	Soil water potential	-25~–10 kPa	-50~–25 kPa	-80~–50 kPa	–	–
	Degree of soil salinization	4.0–8.0 dSm^–1^	8.0–16.0 dSm^–1^	> 16.0 dSm^–1^	–	–

## 2 Materials and methods

### 2.1 Data retrieval strategies and filtering criteria

**Figure 1:**
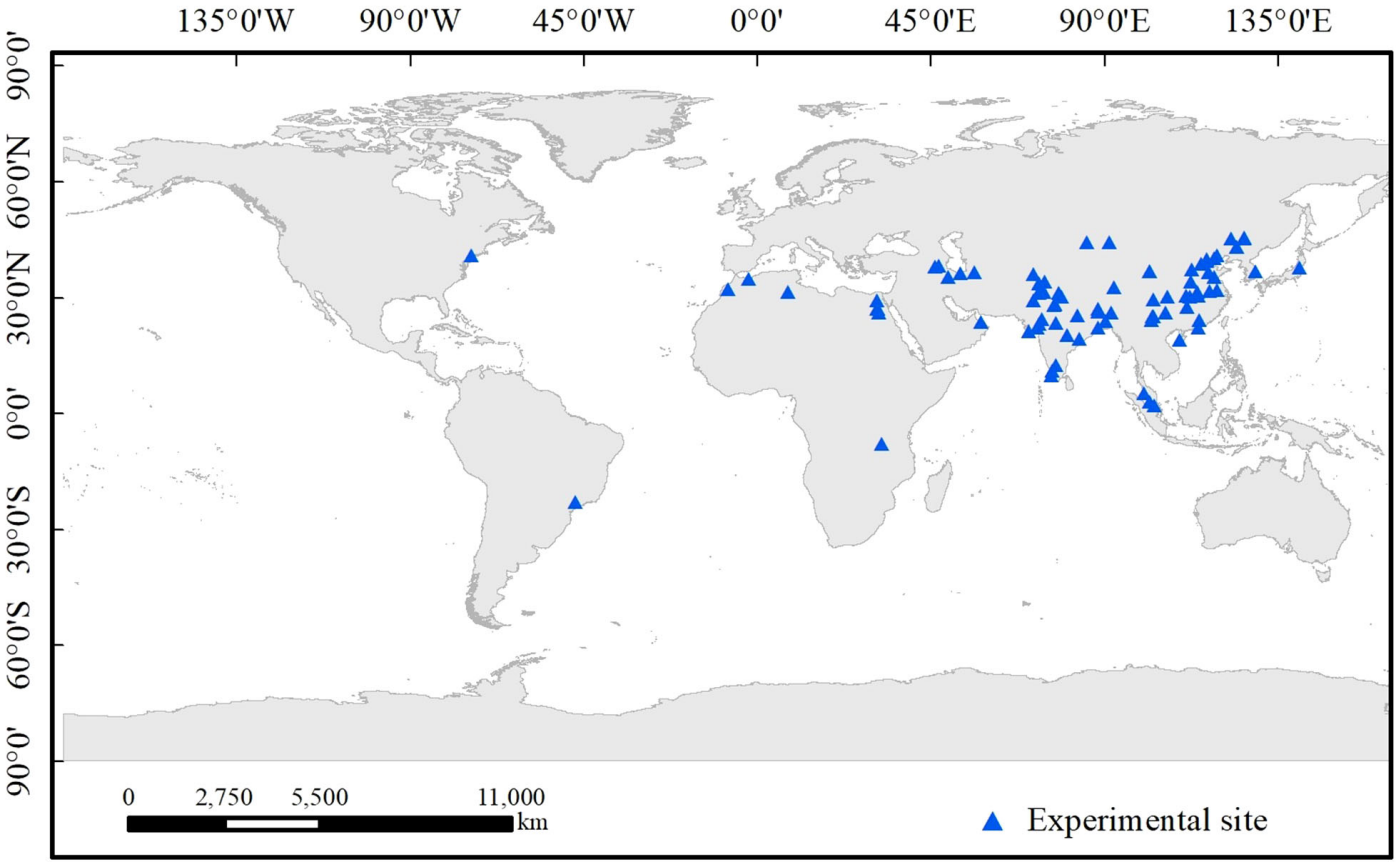
Map of study sites included in the meta-analysis.

The data for the meta-analysis were retrieved from Web of Science (WOS) and the China National Knowledge Infrastructure (CNKI), and the keywords searched included “potassium-solubilizing bacteria”, “yield”, “rhizosphere potassium-solubilizing bacteria”, “microorganisms”, “enzyme activity” and “potassium solubilization”. For the meta-analysis, 102 relevant published studies were collated, including 95 English and 7 Chinese studies (**Figure 1**), containing 846 pairs of paired observations(Among them, 806 pairs of data are from English databases, and 40 pairs of data are from Chinese databases). The selected studies had to satisfy the following requirements: (1) The retrieved variables included the mean, standard deviation (or standard error), and repeat group. (2) All the control and treatment groups belonged to the same ecosystem and experienced the same environmental and growth conditions. (3) Data from only the control and treatment groups inoculated with potassium-solubilizing microorganisms were analyzed in multiple controlled studies with experimental inoculation treatments. (4) The extracted literature was limited to articles published from 2015 to 2025.

### 2.2 Data collection and classification

We also recorded climatic characteristics such as the mean annual temperature (MAT) and mean annual precipitation (MAP), collecting climatic data from the NASA Surface Meteorology and Solar Energy Location site (https://power.larc.nasa.gov/) on the basis of the latitude and longitude of the study site, if not reported in the original study.

Data from all published literature tables were extracted manually, whereas data from figures were extracted via the Get Data (https://getdata.sourceforge.net/download.html) tool.

### 2.3 Meta-data analysis

We used Meta-Win 2.0 software (https://www.metawinsoft.com/) to analyze the data for random effects. The effect value was calculated via the natural logarithm of the response ratio (lnRR) in Equation (2) (Luo et al., 2018), which was then converted to upper and lower 95% confidence intervals (CI) in Equation (4) to evaluate the impact of the KSM on crop yield, soil physicochemical parameters, and crop growth indicators. In several studies, the standard error (SE) in Equation (1) had to be translated to the standard deviation (SD) in Equation (1), which was calculated as (Jian et al., 2016):


(1) $$\text{SD}=\text{SE}\times \sqrt{\text{n}}$$

(2) $$\text{lnRR =ln}\left(\frac{\bar{x_{t}}}{\bar{x_{c}}}\right)$$

The variance of the effect value is as follows:


(3) $$\mathrm{var}(lnRR)=\frac{S_{t}^{2}}{n_{t}{\bar{X}}_{t}^{2}}+\frac{S_{c}^{2}}{n_{c}{\bar{X}}_{c}^{2}}$$

where ${\bar{X}}_{t}$ is the mean of the variable in the treatment group and where ${\bar{X}}_{c}$ is the mean of the variable in the control group in Equation (3). S_t_ is the standard deviation of the variable in the treatment group; S_c_ is the standard deviation of the variable in the control group; n is the sample size; and n_t_ and n_c_ are the sample sizes of the treatment and control groups, respectively in Equation (3). We converted lnRR and its corresponding confidence intervals to the corresponding percentage change.


(4) $$CI=(e^{lnRR}-1)\times 100\%$$

### 2.4 Heterogeneity test and publication bias test

The I^2^ statistic was used to assess heterogeneity, and the test value Q was calculated as follows in Equation (5) and (6) (Huo et al., 2017):


(5) $$\text{Q}=\sum_{\text{i}=1}^{\text{n}}{\text{W}}_{\text{i}}{\left(E_{i}^{2}-{\text{E}}^{2}\right)}^{2}$$

(6) $$I^{2}=[Q-(n-1)]/Q$$

where w_i_ is the weight of group i of data, n is the number of effect sizes, E_i_ is the effect size of the ith group, and E is the average of all data effect sizes in Equation (5). Calculations were made using the random effect model when the heterogeneity test result I^2^ was greater than 50% and the fixed effect model when it was less than 50%. The Rosenthal loss of safety coefficient method was used for the publication bias test. N>5 m+10 (N is the loss of safety coefficient, and m is the sample size) indicates no publication bias. We used AMOS software (IBM SPSS AMOS 20.0.0) to untangle the indirect and direct effects of climate (MAT and MAP), microbial count, soil available potassium content, crop growth indicators, and soil enzyme activities on crop yields (Eisenhauer et al., 2015) via structural equation modeling (SEM). Before modeling, all the data were normalized (ensuring that the data followed a standard normal distribution with a mean value of 0 and standard deviation of 1), and an *a priori* model was designed based on the known effects and relationships among the drivers that had a significant impact on crop yields in our previous analyses. SEM was used instead of multiple regressions since directions can be assigned to several relationships, resulting in multiple explanatory and response variables in one model. Furthermore, the structure of such a model can reveal whether a significant bivariate relationship results from a significant relationship between two given variables and a third variable. Despite using fail-safe N to assess publication bias, our results may still be affected by the underreporting of studies with non-significant or negative findings. While subgroup analysis explored substantial heterogeneity (indicated by I²), residual heterogeneity could remain due to unmeasured factors like experimental duration, KSM strain potency, and unrecorded soil microbial communities, which may limit the generalizability of pooled effects (Jin et al., 2015; Mathur and VanderWeele, 2022).

## 3 Results

### 3.1 Effect of KSM inoculation on crop yield

**Figure 2:**
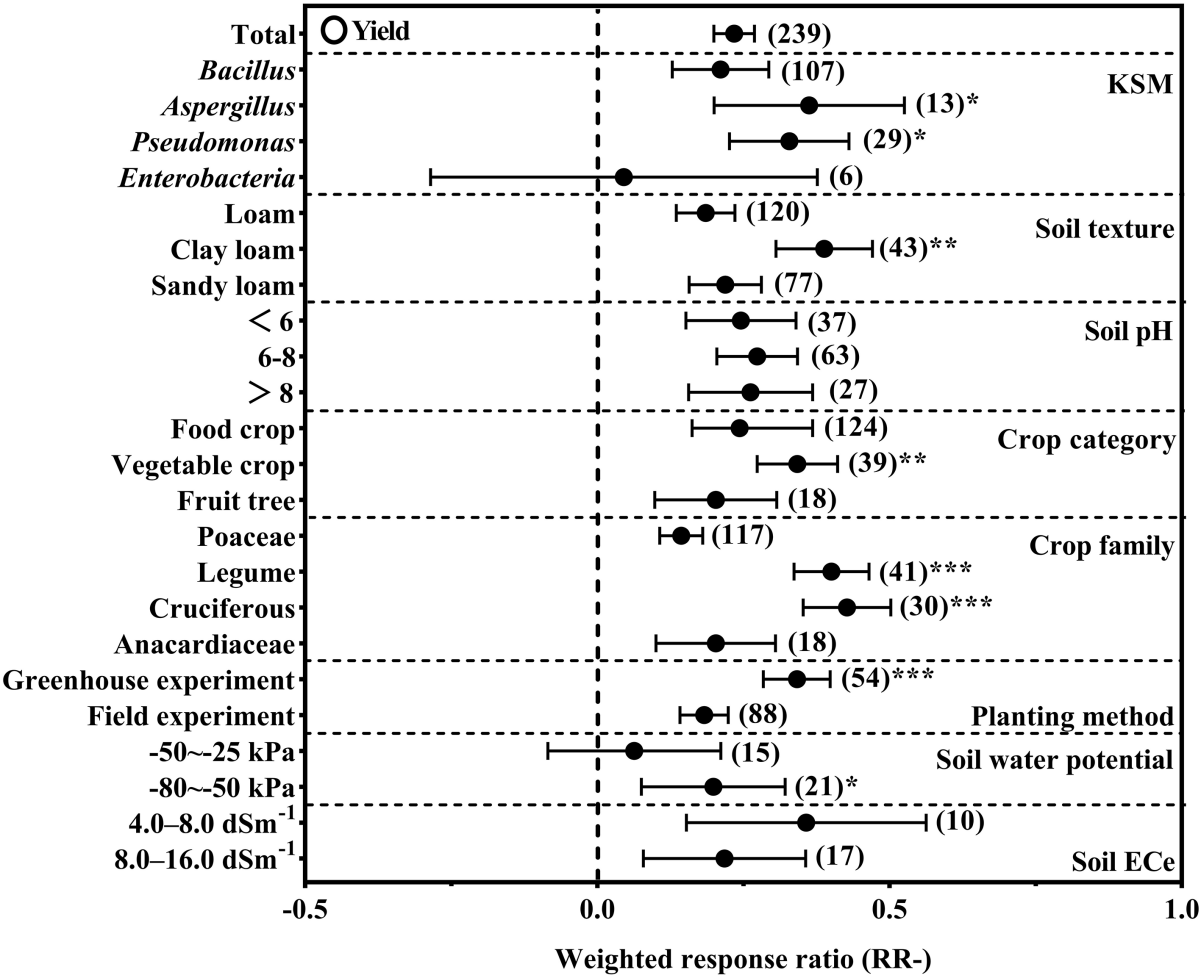
Effect value of KSM inoculation over uninoculated KSM on crop yield (the significant signal means *P<0.05, **P<0.01, ***P<0.001).

The calculation of the effect size on yield revealed that KSM inoculation improved overall crop yield by 23.43% compared with the control, confirming its potential as a yield-enhancing agricultural practice (**Figure 2**). Note: The four KSM genera analyzed (*Aspergillus*, *Pseudomonas*, *Bacillus*, *Enterobacter*) were selected based on two criteria: (1) their high frequency of reporting in existing KSM-related studies (accounting for >70% of eligible literature included in this meta-analysis); (2) their well-documented potassium-solubilizing mechanisms and agricultural application records, ensuring data robustness and practical relevance. Among these genera, *Aspergillus* (+36.27%) and *Pseudomonas* (+32.86%) were consistently demonstrated to outperform *Bacillus* and *Enterobacter* in boosting crop yields (P<0.05).

Soil texture emerged as a key modulating factor: clay loam soil (+38.82%) showed a significantly stronger yield response to KSM than sandy loam and loam (P<0.05), while soil pH had no substantial impact. Crop type and family also drove marked differences: vegetable crops (+34.25%) exhibited the greatest yield increase relative to food crops and fruit trees (P<0.05), with cruciferous (+42.74%) and leguminous (+40.08%) crops outperforming Poaceae and Anacardiaceae (P<0.05). Additionally, greenhouse experiments (+34.17%) yielded better results than field trials (P<0.05), whereas soil water potential and EC values did not induce significant variations in yield response.

### 3.2 Effect of KSM inoculation on soil physicochemical properties

#### 3.2.1 Effect of KSM inoculation on soil nutrients and pH

Compared with the control, KSM inoculation significantly modified soil potassium status and pH, with soil total potassium and available potassium increasing by 18.28% and 28.91%, respectively, and soil pH decreasing by 18.10% (**Supplementary Figures S2A, B**).

Notably, the efficacy of KSM in boosting soil available potassium varied markedly by biological and environmental factors: *Enterobacter* (+39.22%) showed a significantly stronger capacity to mobilize available potassium than *Bacillus* and *Pseudomonas* (P<0.05), while loam soil (+32.25%) outperformed clay loam and sandy loam in this regard (P<0.05). Crop-related factors also drove substantial differences: vegetable crops (+50.4%) and Poaceae (+32.71%) exerted the greatest positive effects on soil potassium availability among crop types and families, respectively (P<0.05). In contrast, soil pH had no substantial impact on KSM-mediated available potassium augmentation, indicating that KSM can stably improve potassium availability across diverse soil acid-base conditions.

For soil pH regulation, all tested KSM genera induced negative effects, though with no significant differences between them. Soil texture and initial pH modulated this effect: loam (–18.32%) and clay loam (–25.06%) showed stronger pH reduction than sandy loam, and KSM inoculation at pH>8 (–25.38%) had a significantly greater acidifying effect than at pH 6–8 (P<0.05), suggesting that KSM are more effective at lowering pH in alkaline soils.

#### 3.2.2 Effect of KSM inoculation on soil enzyme activity

KSM inoculation significantly enhanced the activities of both antioxidant-related and metabolism-related enzymes, with peroxidase (+41.0%) and cellulase (+147.3%) showing the highest enhancement rates, respectively (**Supplementary Figure S2A, C**).

For antioxidant-related enzymes, superoxide dismutase activity trended positive in sandy loam and loam but decreased in clay loam, while peroxidase was beneficial only in loam soil. In contrast, catalase activity showed clear strain and texture specificity: *Pseudomonas* (+50.92%) was significantly more effective than *Bacillus* (P<0.05), and clay loam/loam supported positive responses whereas sandy loam showed a negative effect. Consistently across all antioxidant enzymes, KSM promoted activity at soil pH<6 but suppressed it at pH>8 relative to the control, highlighting soil pH as a key regulator of KSM-induced antioxidant defense.

Metabolism-related enzymes showed universal enhancement, with protease (+58.9%), chitinase (+62.7%), and cellulase (+147.3%) exhibiting the strongest activity increases, indicating that KSM play a critical role in accelerating soil organic matter decomposition and nutrient cycling. Sucrase showed the weakest response (+13.6%), suggesting that KSM have enzyme-specific regulatory effects on soil metabolic processes.

### 3.3 Effect of KSM inoculation on crop growth

#### 3.3.1 Effect of KSM inoculation on crop potassium content

**Figure 3:**
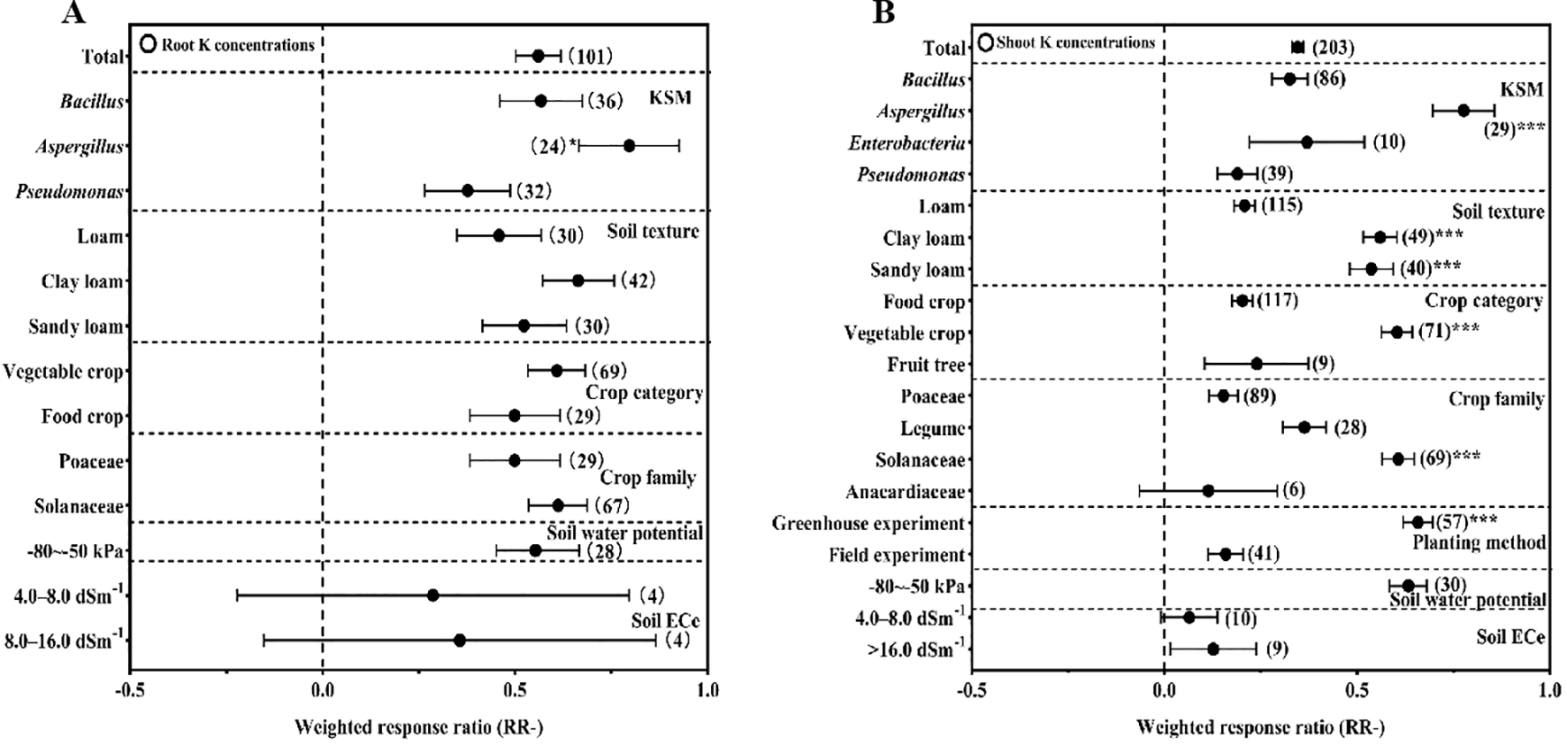
Effect of different conditions on K concentrations in roots **(A)** and in shoots **(B)** under inoculation with KSM (the significant signal means *P<0.05, **P<0.01, ***P<0.001).

KSM inoculation significantly enhanced potassium uptake in crops, with root K concentration and shoot K concentration both showing marked increases relative to the control (**Figures 3A, B**).

For root K concentration, *Aspergillus* (+79.64%) was the most effective genus, outperforming *Bacillus* and *Pseudomonas* (P<0.05). Notably, the effect values for root K concentration were consistently positive across soil textures, crop types, crop families, and soil ECe conditions, with no significant variations between subgroups—indicating that KSM promotes root potassium accumulation stably across diverse contexts.

Shoot K concentration was increased by 31.11% overall, with *Aspergillus* (+77.65%) again outperforming *Enterobacter*, *Bacillus*, and *Pseudomonas* (P<0.05). Soil texture significantly modulated this effect: clay loam (+55.99%) and sandy loam (+53.71%) were more effective than loam (P<0.05). By crop category, vegetable crops (+60.38%) showed a stronger response than food crops and fruit trees (P<0.05), and Solanaceae (+60.67%) outperformed other crop families (P<0.05). No significant differences in shoot K concentration were detected under various soil ECe conditions.

#### 3.3.2 Effect of KSM inoculation on growth indices

Compared with the control, KSM significantly increased all measured crop growth indices, with dry root weight (+59.21%) and total chlorophyll content (+56.06%) showing the most dramatic improvements, followed by leaf area (+44.67%) and root fresh weight (+40.79%) (**Supplementary Figure S3A**).

**Figure 4:**
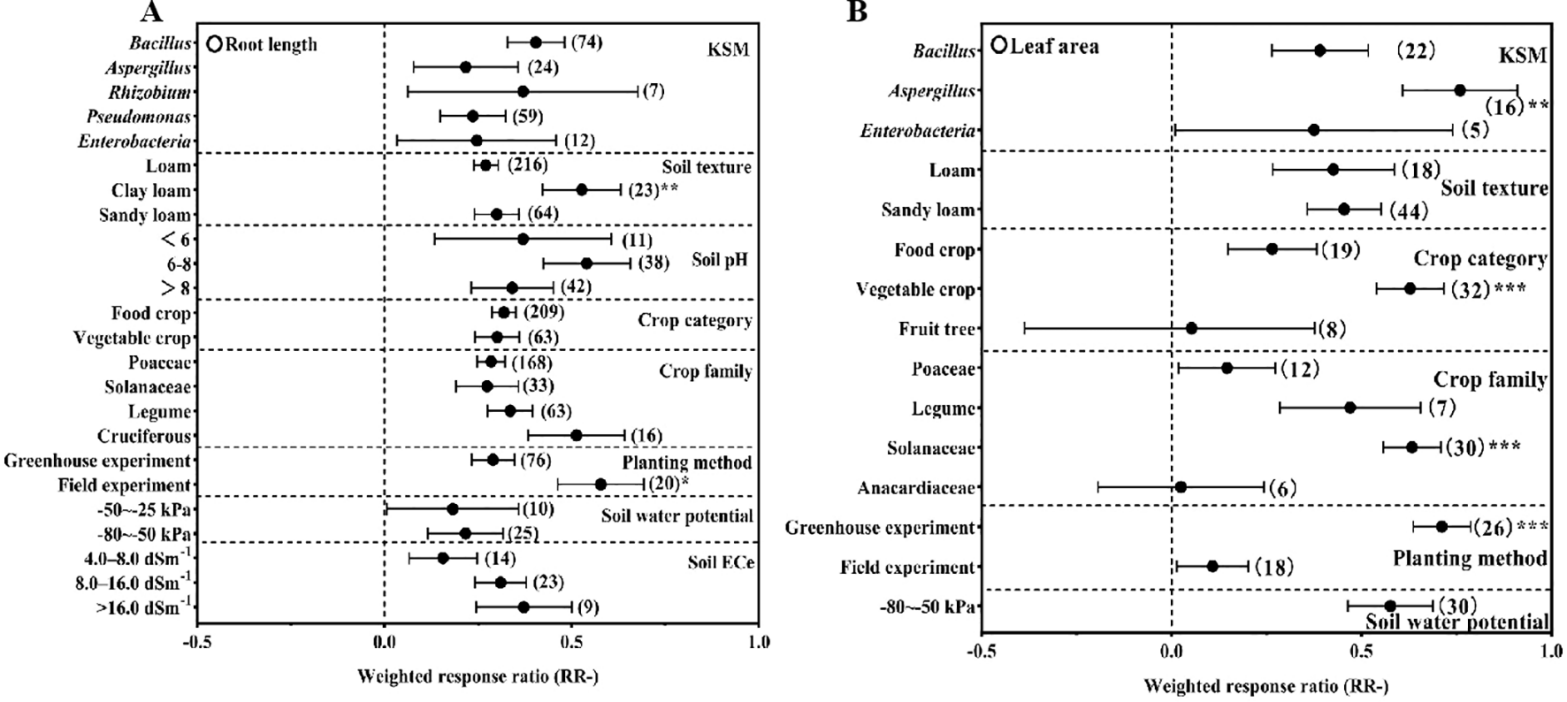
Effect of different conditions on root length **(A)**, leaf area **(B)** under inoculation with KSM (the significant signal means *P<0.05, **P<0.01, ***P<0.001).

For root length (**Figure 4A**), KSM exerted positive effects across all subgroups, with clay loam soil (+52.71%) and field experiments (+57.20%) standing out as the most favorable conditions (P<0.05). Notably, plant height responded strongly to *Aspergillus* inoculation (+54.33%), which was significantly more effective than other genera (P<0.05); sandy loam soil (+36.71%) and vegetable crops (+43.30%), particularly solanaceae (+47.11%), also showed enhanced plant height under KSM treatment (P<0.05). In contrast, soil pH had no significant influence on plant height. Greenhouse experiments (+49.86%) outperformed field trials in promoting plant height (P<0.05), and the optimal soil water potential range was –80 to –50 kPa (+55.64%, P<0.05).

Leaf area was most notably increased by *Aspergillus* (+76.00%, P<0.05), with vegetable crops (+62.89%) and solanaceae (+63.37%) again showing stronger responses than other crop types/families (P<0.05, **Figure 4B**). Greenhouse conditions (+71.24%) also facilitated a greater increase in leaf area compared to field settings (P<0.05), while soil texture and pH had no significant differential effects.

### 3.4 Effect of KSM inoculation on the K content in soil and crops in relation to environmental factors

**Figure 5:**
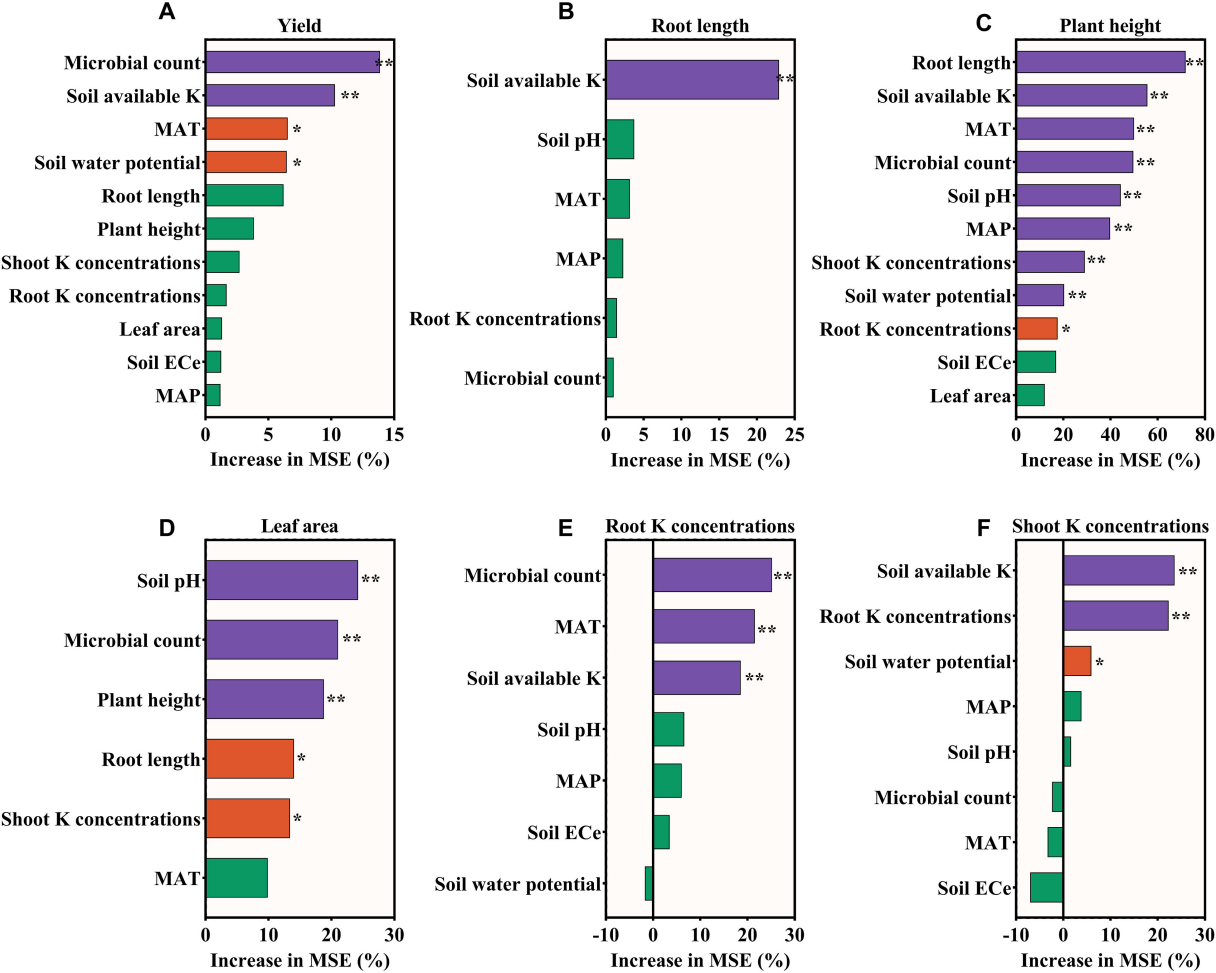
Distribution of the variable importance for **(A)** Yield, **(B)** Root length, **(C)** Plant height, **(D)** Leaf area, **(E)** Root K concentrations, and **(F)** Shoot K concentrations random forest models including the different Microbial count, climatic conditions, soil properties and Growth indicators as variable, the significant signal means *P<0.05, **P<0.01.

A key chain emerged from the correlation analysis (**Figure 5**): soil available potassium was significantly and positively correlated with both crop shoot K concentrations (P<0.05) and root K concentrations (P<0.05), while root K concentrations further positively linked to shoot K concentrations (P<0.05). Critically, both soil available potassium and crop shoot K concentrations showed direct positive correlations with crop yield (P<0.05), highlighting that KSM’s yield-enhancing effect is likely mediated through improved soil K availability and subsequent crop K uptake.

### 3.5 Relationships between effect sizes of inoculation with KSM on yield and other growth indicators

**Figure 6:**
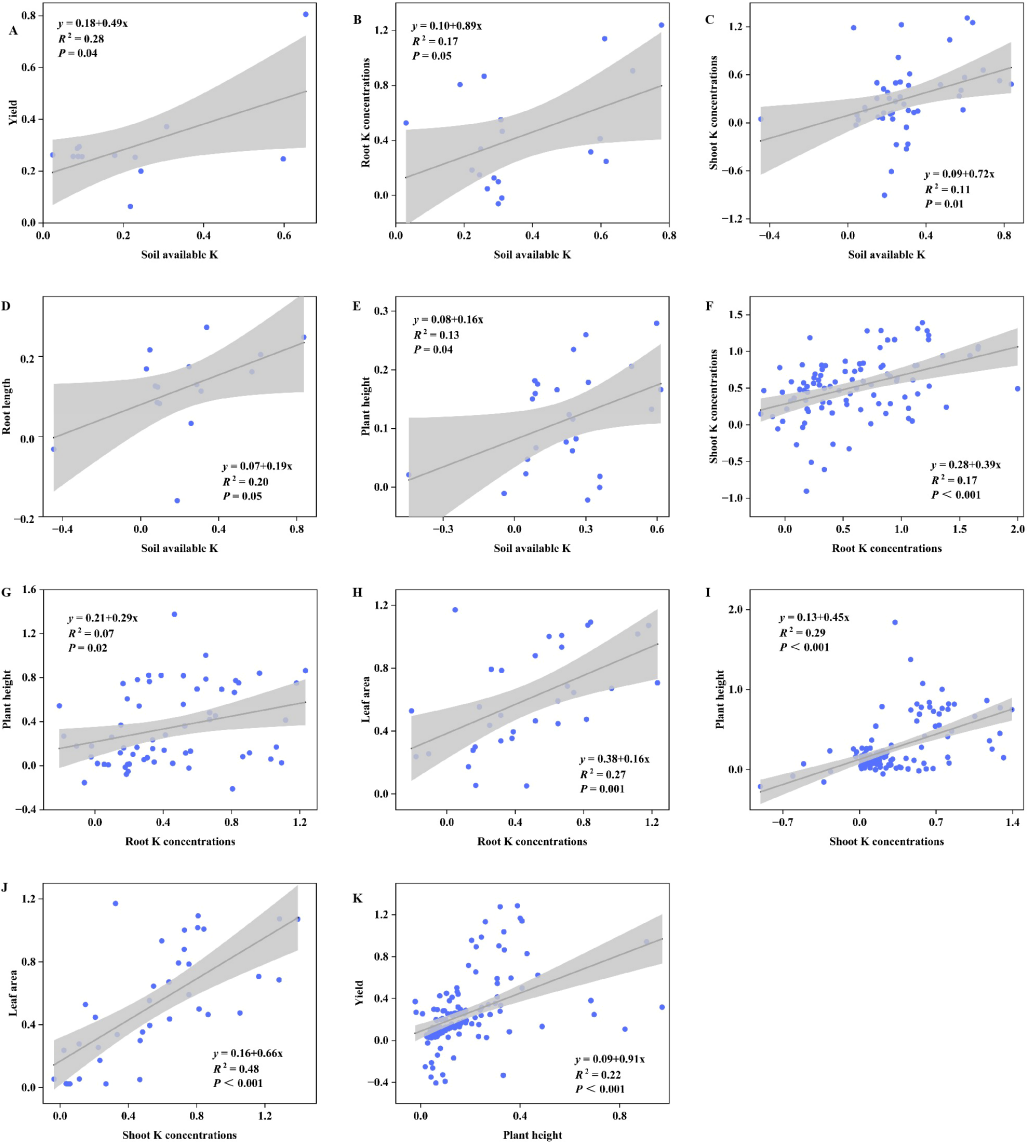
Relationship between the effect size of soil available K and **(A)** yield, B) root K concentrations, **(C)** shoot K concentrations, **(D)** root length and **(E)** plant height; root K concentrations and **(F)** shoot K concentrations, **(G)** plant height and **(H)** leaf area; shoot K concentrations and **(I)** plant height, **(G)** leaf area; plant height and **(K)** yield. Reported statistical results from two-sided linear regressions. Gray areas represent 95%Cl.

The results of the random forest analysis are presented in **Figure 6**. We assigned significance to factors influencing yield and other growth indicator changes by considering those with a significance level greater than 0.1. For yield, the key control factor is microbial count and soil available K. For root length, the key control factor is soil available K. For plant height, the key control factors are root length, soil available K, MAT, microbial count, soil pH, MAP, shoot K concentrations, and soil water potential. For leaf area, significant control factors included soil pH, microbial count, and plant height. For root K concentrations, significant control factors included microbial count, MAT, and soil available K. For shoot K concentrations, the significant control factors included soil available K and root K concentrations.

### 3.6 Impact of microbial count, MAT, and MAP on crop yield following KSM inoculation

**Figure 7:**
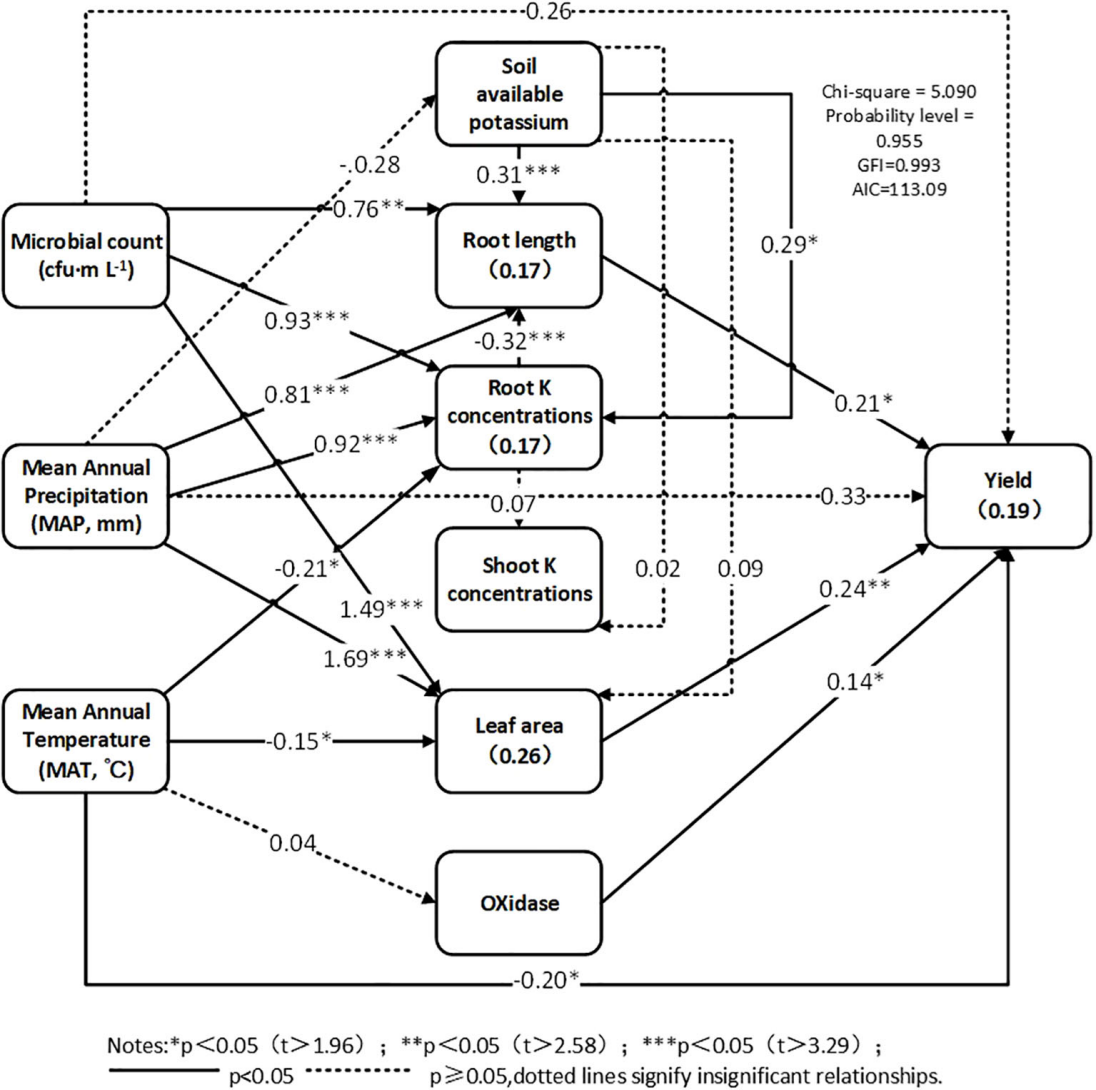
Structural equation modeling (SEM) describing the direct and indirect effects of predictor variables on effect size of field.

Structural equation modeling (SEM) was used to investigate how the various explanatory variables and their interactions influenced crop yield under KSM inoculation (**Figure 7**). The final structural equation model explained 19% of the overall variation in crop yield (χ^2^ = 5.09; P = 0.955, AIC = 113.09). The microbial count, MAP, MAT, soil available potassium, root K concentration, root length, leaf area, and oxidase were the most crucial factors affecting crop yield, both directly and indirectly. Overall, the microbial count indirectly affects crop yield by altering root length (path coefficient = 0.76), root K concentration (path coefficient = 0.93), and leaf area (path coefficient = 1.49). MAP indirectly affects crop yield by influencing root length (path coefficient = 0.81), root K concentration (path coefficient = 0.92), and leaf area (path coefficient = 1.69). The MAT had an indirect effect (path coefficient = 0.81), and the root K concentration (path coefficient = 0.92) and leaf area (path coefficient = 1.69) were affected. MAT also indirectly improved crop yield by influencing root K concentrations (path coefficient =–0.21) and leaf area (path coefficient =–0.15). Soil available potassium indirectly affected crop yield by influencing root length (path coefficient = 0.31) and root K concentrations (path coefficient = 0.29). The root K concentrations also indirectly influenced crop yield because of their impact on root length (path coefficient =–0.32). Root length had a direct positive effect on crop yield (path coefficient = 0.21), and leaf area had a direct positive effect on crop yield (path coefficient = 0.24). Oxidase had a direct positive effect on crop yield (path coefficient = 0.14). These results confirm the potential of KSM to increase crop production through inoculation.

## 4 Discussion

### 4.1 Effects of KSM on different microbial genera and soil factors

Our results showed that KSM significantly increased crop yield, soil available K, and crop potassium uptake and growth, confirming their role in bioweathering potassium-containing minerals and mediating soil potassium transformation (Chen et al., 2022). Elevated root and shoot K concentrations further promote crop growth and yield, which aligns with Olaniyan who emphasized that enhanced potassium nutrition improves root vigor and photosynthetic efficiency (Olaniyan et al., 2022).

Crop yield and development varied with KSM genus, soil texture, and other contextual factors. Among tested microorganisms, *Aspergillus* showed the strongest overall effect on crop K content and yield, outperforming *Bacillus* and *Pseudomonas*. This superiority stems from its synergistic mechanisms: it secretes multiple extracellular organic acids (e.g., oxalic acid) to disrupt potassium-bearing mineral lattices and releases phosphatases/glucosidases (Ashrafi-Saiedlou et al., 2024), while producing phytohormones and iron transporters to optimize root physiological functions (Kumawat et al., 2024). Unlike *Bacillus* (which primarily relies on acidolysis), *Aspergillus* also improves rhizosphere microecology and soil aggregation (Imran et al., 2021), making it a key candidate for microbial preparation development and potash fertilizer efficiency improvement.

With respect to soil texture, clay loam showed the strongest yield response to KSM, which is attributed to the unique improvement effect of KSM on this soil type. Clay loam typically suffers from poor aeration that hinders root nutrient uptake (Hsiao et al., 2018), but KSM inoculation maintains stable available potassium levels during the crop reproductive period (Singh et al., 2022) and produces extracellular polysaccharides to promote soil aggregation (Wang et al., 2022). This structural improvement increases aeration and nutrient diffusion, enhancing root K uptake and root length (a trend consistent with the significant increase in root K concentration and length observed in clay loam). These findings indicate that KSM can targeted alleviate the limitations of clay loam soils, providing a solution for yield improvement in such agricultural systems.

### 4.2 Effects of KSM under different crop classifications, field management practices, and stress types

With respect to crop classification, our synthesis indicated that vegetable crops responded more positively to KSM than other crop types, which can be explained by their biological traits: most vegetables have short growth cycles, high potassium demand, and quality formation closely linked to potassium nutrition (Zoerb et al., 2014). Compared with cereals, vegetables such as sugar beet exhibit higher K mobilization efficiency per unit root length (El Dessougi et al., 2002; Wang et al., 2011), making them more sensitive to KSM-mediated potassium supplementation. This finding provides a basis for targeted KSM application—prioritizing vegetable crops in agricultural production to maximize the return on microbial inoculation.

With respect to field management, greenhouse conditions significantly enhanced KSM efficacy relative to field trials, as the controlled temperature, humidity, and light optimize both crop growth and microbial survival (Shamshiri et al., 2018; Farvardin et al., 2024). This suggests that KSM application should be paired with appropriate environmental regulation to fully exploit its potential.

Under stress types, KSM consistently improved crop performance across drought and salinity gradients, which is achieved through dual mechanisms: supplementing potassium to maintain physiological functions (e.g., photosynthesis, enzyme activity) (Munsif et al., 2022) and enhancing stress defense systems (e.g., increasing antioxidant enzyme activity) (Feng et al., 2019). For salinity stress, KSM also helps maintain K^+^/Na^+^ balance (Romero-Munar and Aroca, 2023), further confirming its role in improving crop stress resilience.

### 4.3 Key influences of the KSM on improving crop yields

This study demonstrated a significant linear correlation among soil available potassium, root/shoot potassium concentrations, and crop yield following KSM inoculation, consistent with previous findings (Shen et al., 2016; Ai et al., 2022). The increase in yield can be attributed to a well-defined physiological pathway initiated by KSM-mediated potassium solubilization.

KSM enhances the availability of soil potassium, leading to elevated potassium uptake by roots and subsequent translocation to shoots (Chen et al., 2021). The increased potassium levels stimulate root growth, including surface area, vigor, and biomass, which further expands nutrient absorption capacity and improves water retention (Sustr et al., 2019). Simultaneously, potassium promotes shoot development by facilitating cell expansion, leaf area enlargement, and photosynthetic efficiency (Hu et al., 2022; Ding et al., 2024).

Our SEM analysis revealed a multi-level regulatory network of KSM on yield, with soil available potassium and microbial count as core indirect drivers, and root length/leaf area as direct effectors. Soil available potassium promotes root tip cell division and elongation by regulating growth hormone transport (Sheng, 2005), while microbial count modulates root traits and nutrient availability (Sun et al., 2023), thereby establishing a coherent pathway from potassium activation to root growth and, ultimately, yield increase. Climate factors also play a role: MAP improves soil moisture to enhance root uptake (Li et al., 2023a), while MAT shows dual effects—moderate temperatures promote metabolism, but extreme temperatures inhibit root K uptake (Jacobson et al., 1957), highlighting the need for temperature adaptation in KSM application.

These findings have important implications for sustainable agriculture: KSM can reduce reliance on mineral potash fertilizers by improving soil potassium use efficiency, particularly in clay loam and vegetable cropping systems. However, practical application must consider contextual limitations, for example, KSM efficacy may decline in highly saline soils due to microbial survival constraints. Future research should focus on optimizing inoculation strategies and developing climate-adapted KSM formulations to enhance applicability across diverse agricultural environments.

## 5 Conclusions

This meta-analysis clarified the efficacy and regulatory mechanisms of KSM across diverse environments, filling the gap between local trials and global application guidelines. Key findings: (1) KSM inoculation significantly improved multiple agronomic parameters via a sequential process initiated by boosting soil available potassium, followed by enhanced plant potassium assimilation, and culminating in yield increase.; (2) Structural equation modeling identified microbial count and soil available K as core indirect drivers, with root length/leaf area as direct effectors; (3) *Aspergillus* was the most effective genus, with optimal responses in clay loam, vegetable crops, and greenhouse conditions. These findings provide a data-driven basis for reducing mineral potash fertilizer reliance—practically, we recommend targeted inoculation of Aspergillus in clay loam vegetable fields, combined with greenhouse environmental regulation. In summary, KSM is a potent biological strategy for sustainable agriculture, and our work offers actionable pathways for its optimized application.

## Data Availability

The original contributions presented in the study are included in the article/**Supplementary Material**. Further inquiries can be directed to the corresponding author/s.
