# Supplementary figures and images for: Multi-environment meta-analysis reveals the mechanism of action of potassium-solubilizing microorganisms on crop yields
Source: Front Plant Sci. 2025 Nov 3;16:1659478. doi: 10.3389/fpls.2025.1659478 (PMC12620454; doi:10.3389/fpls.2025.1659478)

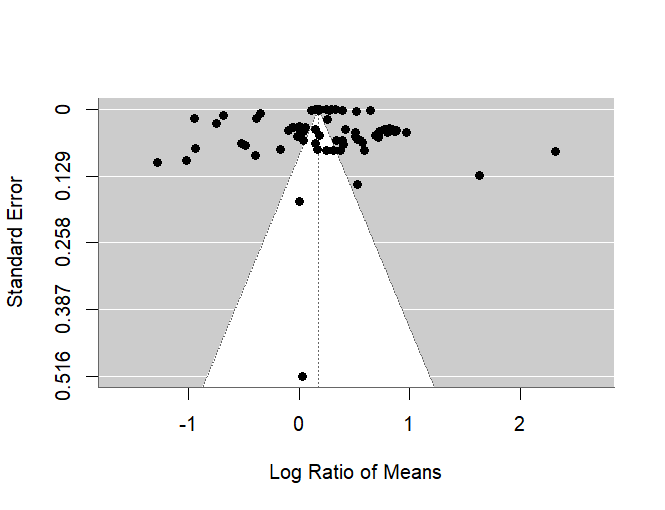

Supplement: Supplementary file 2 [file DataSheet1.zip › Funnel chart/CAT.png]

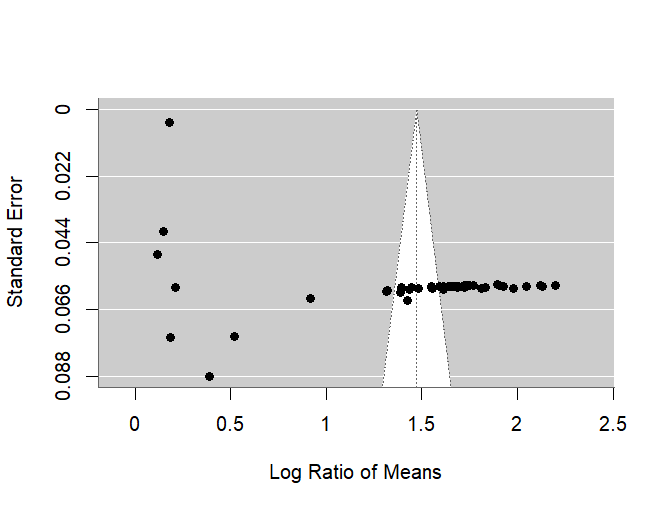

Supplement: Supplementary file 2 [file DataSheet1.zip › Funnel chart/Cellulase.png]

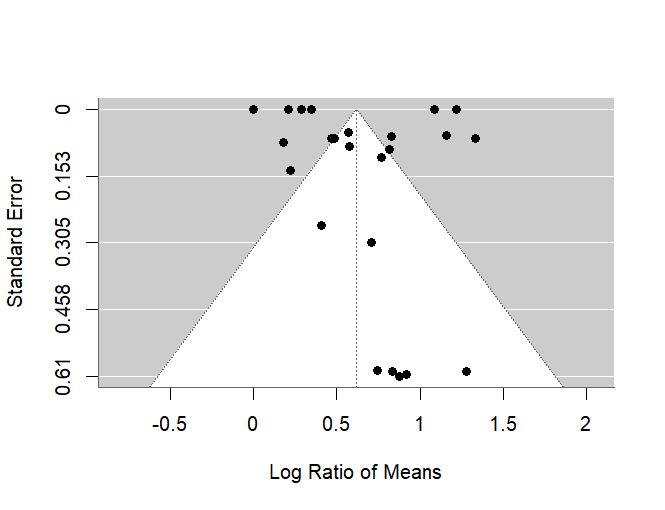

Supplement: Supplementary file 2 [file DataSheet1.zip › Funnel chart/Chitinase.png]

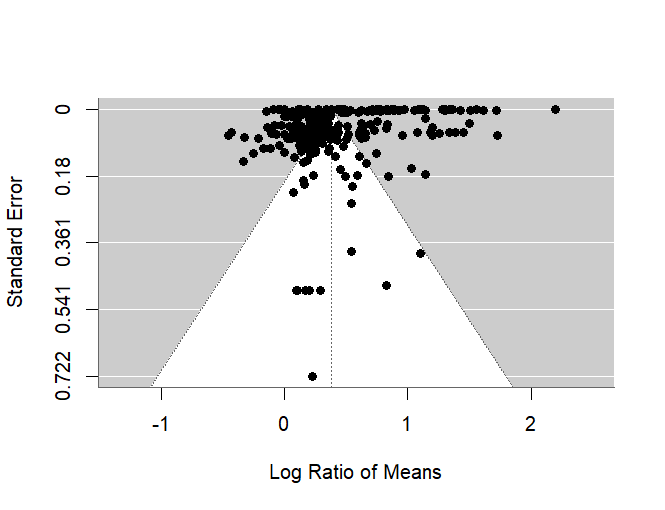

Supplement: Supplementary file 2 [file DataSheet1.zip › Funnel chart/Dry plant weight.png]

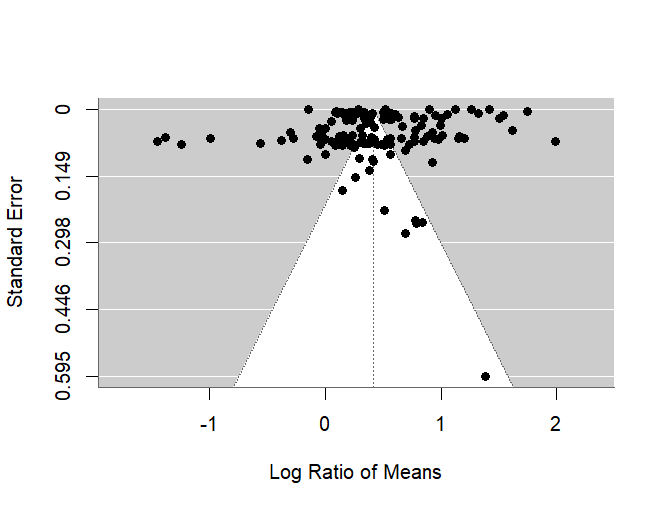

Supplement: Supplementary file 2 [file DataSheet1.zip › Funnel chart/Dry root weight.png]

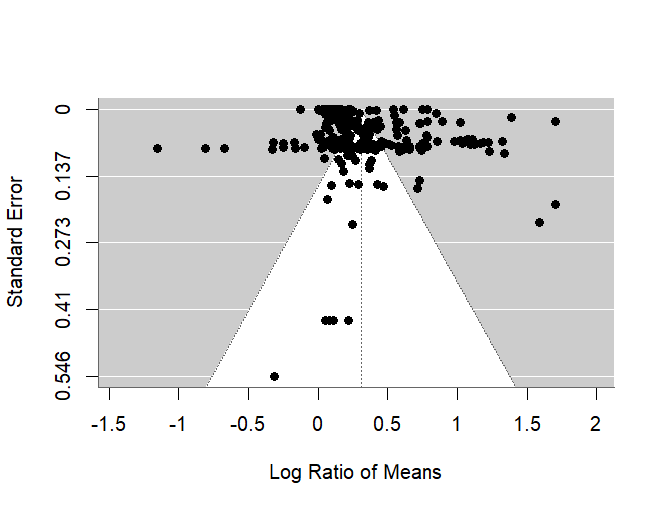

Supplement: Supplementary file 2 [file DataSheet1.zip › Funnel chart/Fresh plant weight.png]

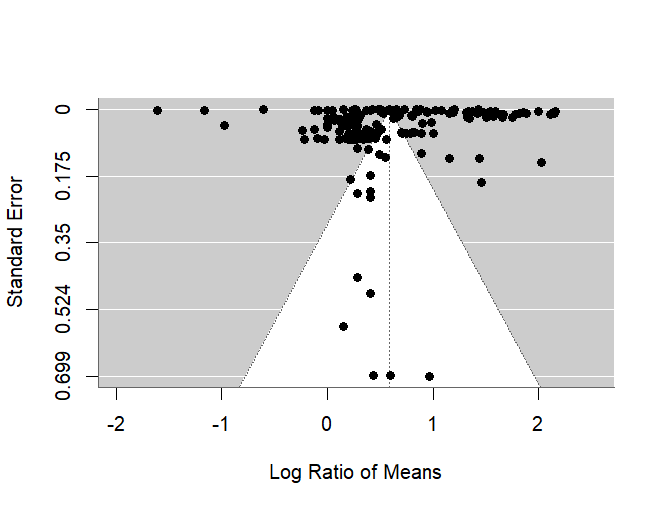

Supplement: Supplementary file 2 [file DataSheet1.zip › Funnel chart/Fresh root weight.png]

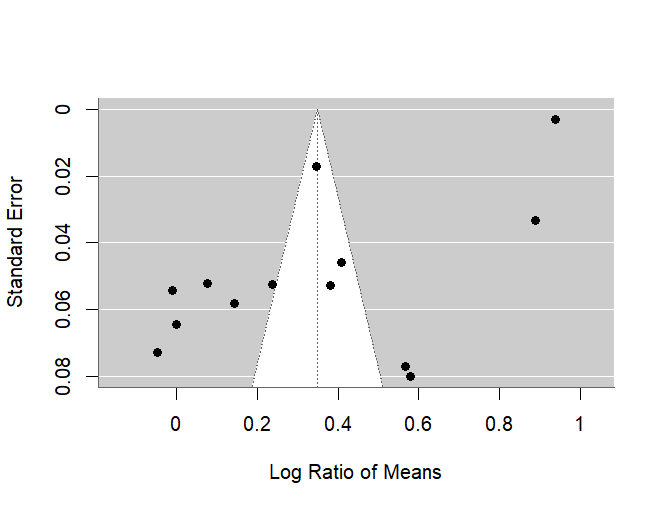

Supplement: Supplementary file 2 [file DataSheet1.zip › Funnel chart/Invertase.png]

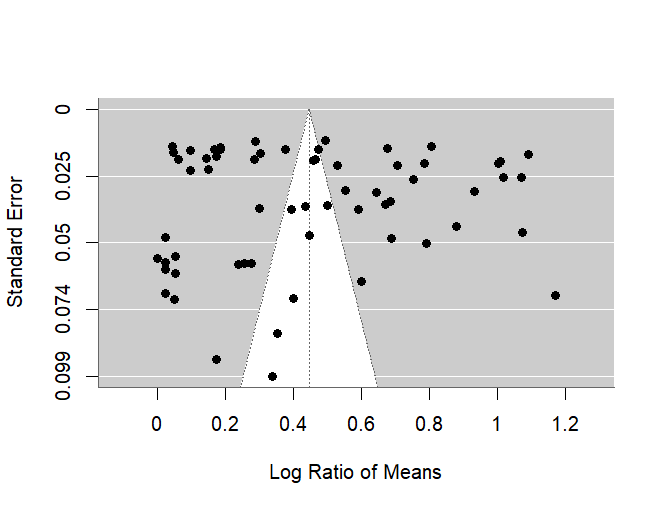

Supplement: Supplementary file 2 [file DataSheet1.zip › Funnel chart/Leaf area.png]

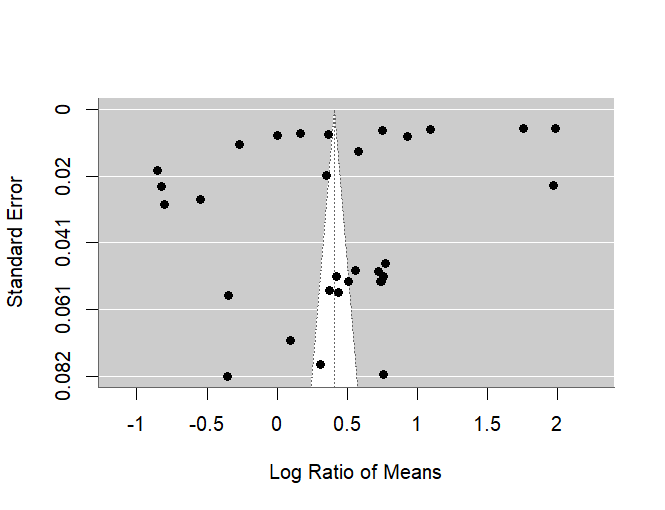

Supplement: Supplementary file 2 [file DataSheet1.zip › Funnel chart/POD.png]

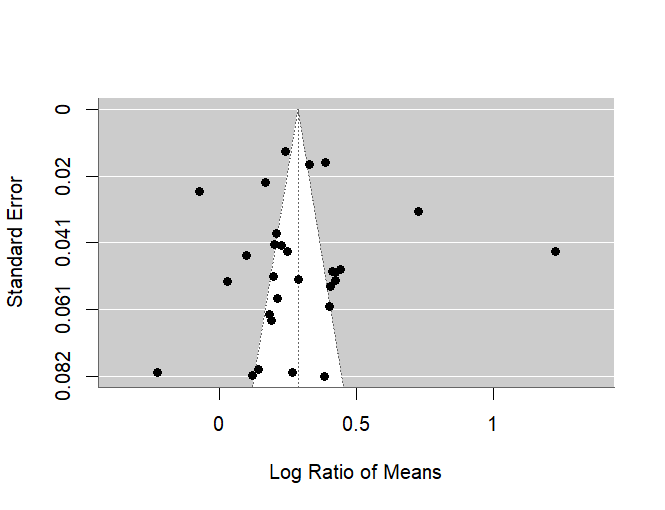

Supplement: Supplementary file 2 [file DataSheet1.zip › Funnel chart/Phosphatase.png]

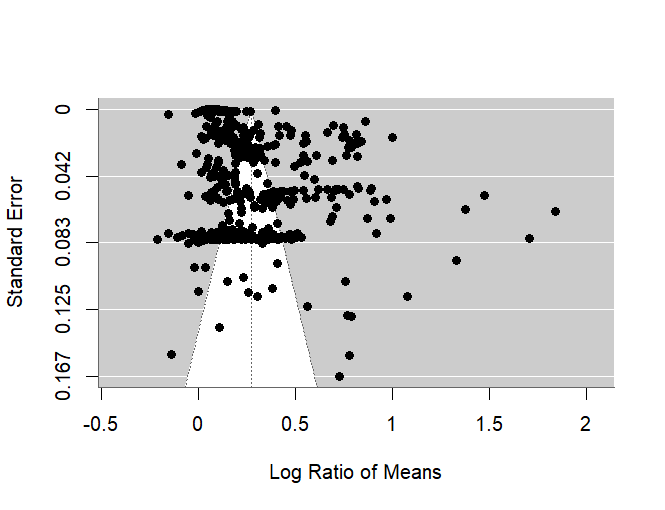

Supplement: Supplementary file 2 [file DataSheet1.zip › Funnel chart/Plant height.png]

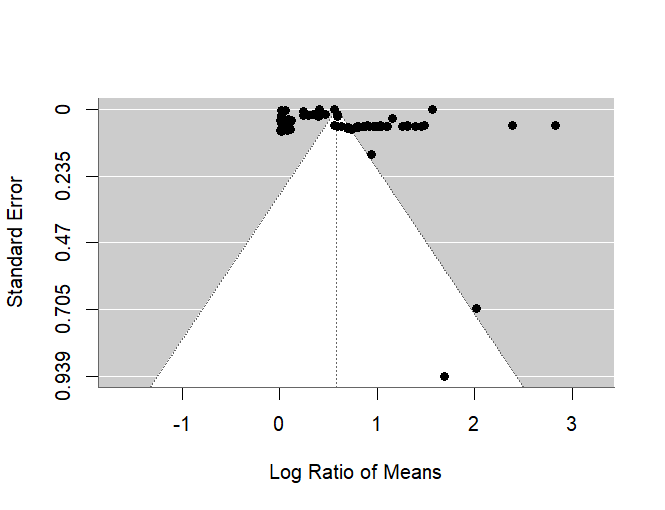

Supplement: Supplementary file 2 [file DataSheet1.zip › Funnel chart/Protease.png]

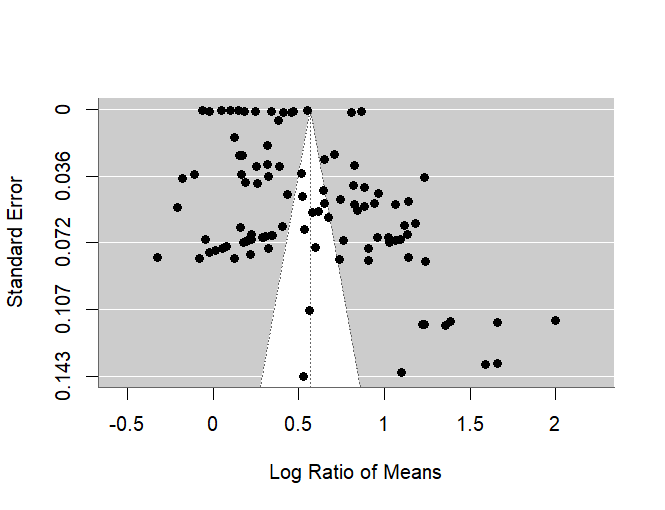

Supplement: Supplementary file 2 [file DataSheet1.zip › Funnel chart/Root K concentrations.png]

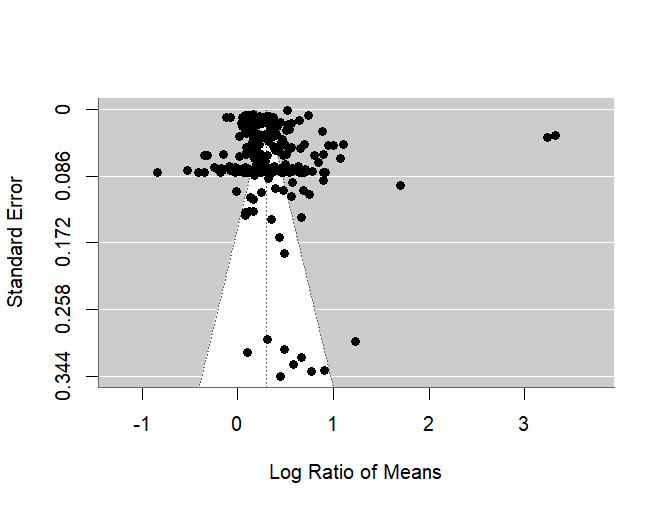

Supplement: Supplementary file 2 [file DataSheet1.zip › Funnel chart/Root length.png]

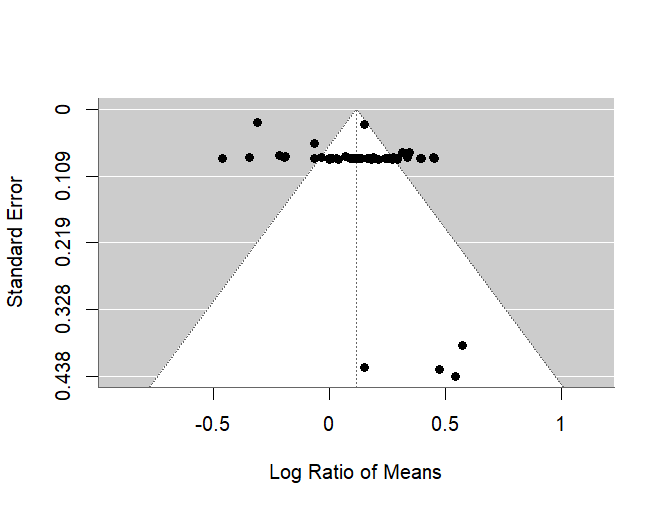

Supplement: Supplementary file 2 [file DataSheet1.zip › Funnel chart/SOD.png]

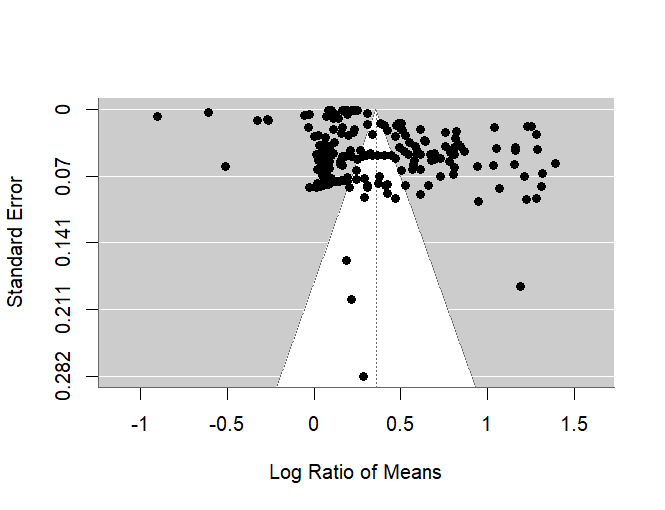

Supplement: Supplementary file 2 [file DataSheet1.zip › Funnel chart/Shoot K concentrations.png]

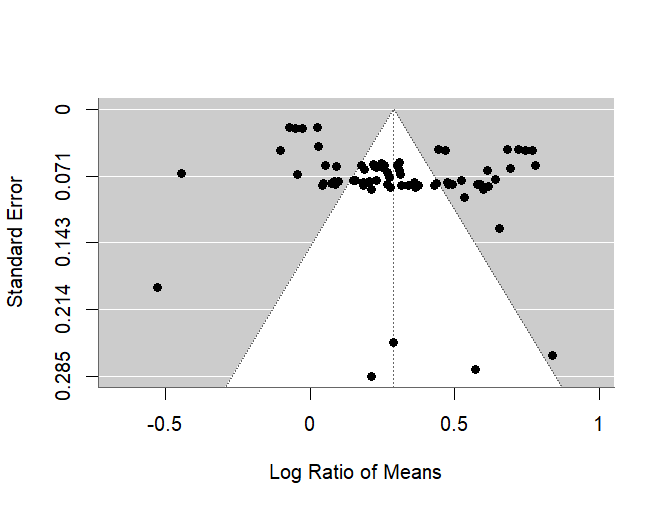

Supplement: Supplementary file 2 [file DataSheet1.zip › Funnel chart/Soil available potassium.png]

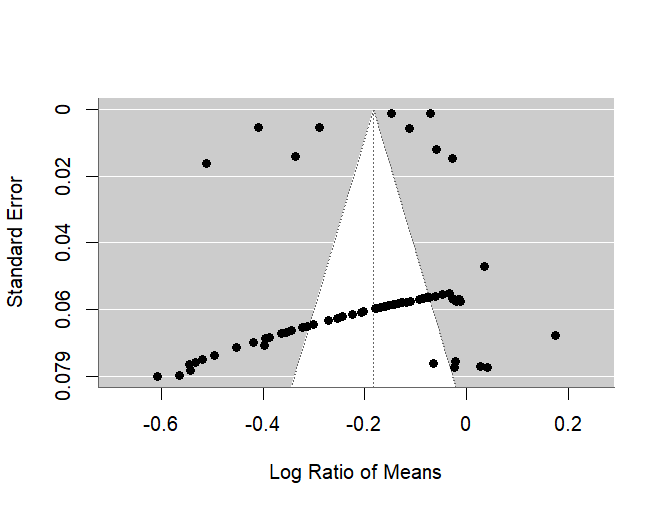

Supplement: Supplementary file 2 [file DataSheet1.zip › Funnel chart/Soil pH.png]

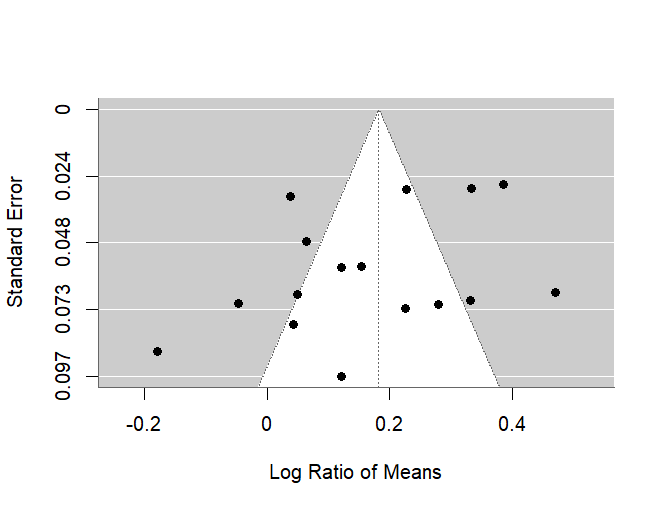

Supplement: Supplementary file 2 [file DataSheet1.zip › Funnel chart/Soil total potassium.png]

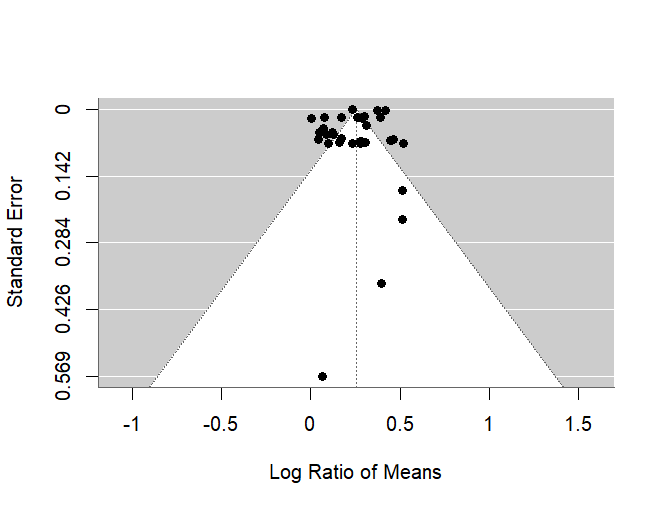

Supplement: Supplementary file 2 [file DataSheet1.zip › Funnel chart/Stem thickness.png]

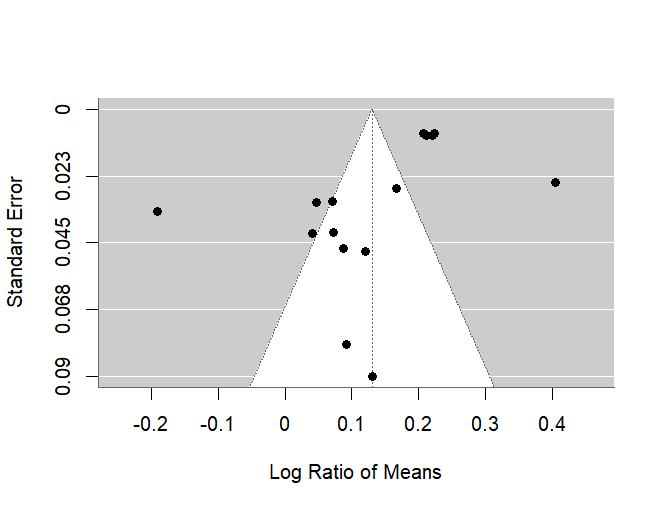

Supplement: Supplementary file 2 [file DataSheet1.zip › Funnel chart/Sucrase.png]

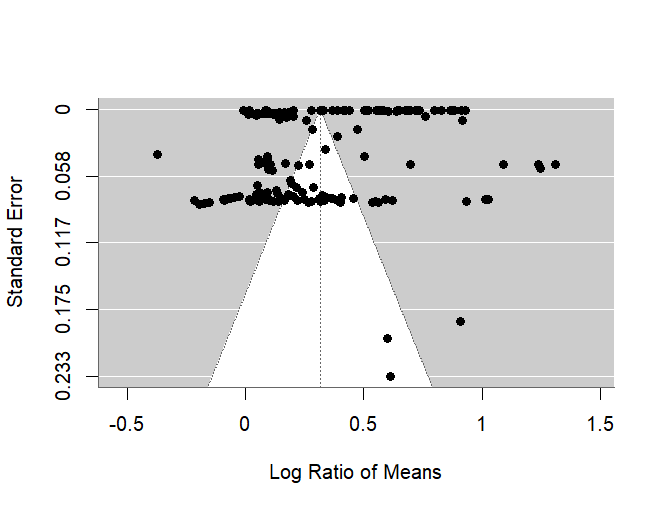

Supplement: Supplementary file 2 [file DataSheet1.zip › Funnel chart/Total chlorophyll.png]

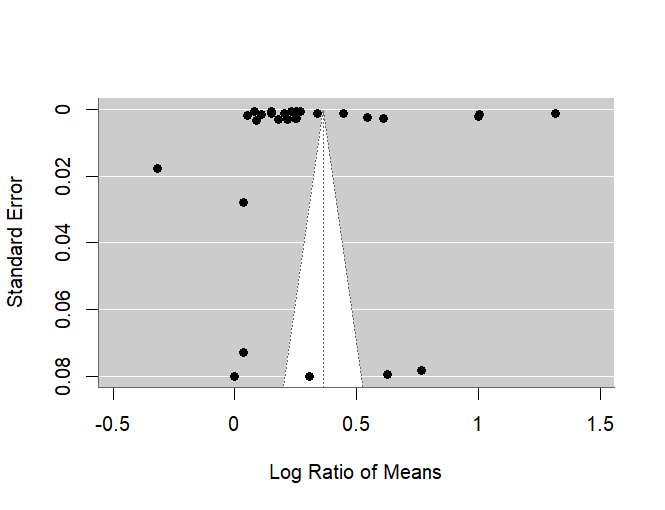

Supplement: Supplementary file 2 [file DataSheet1.zip › Funnel chart/Urease.png]

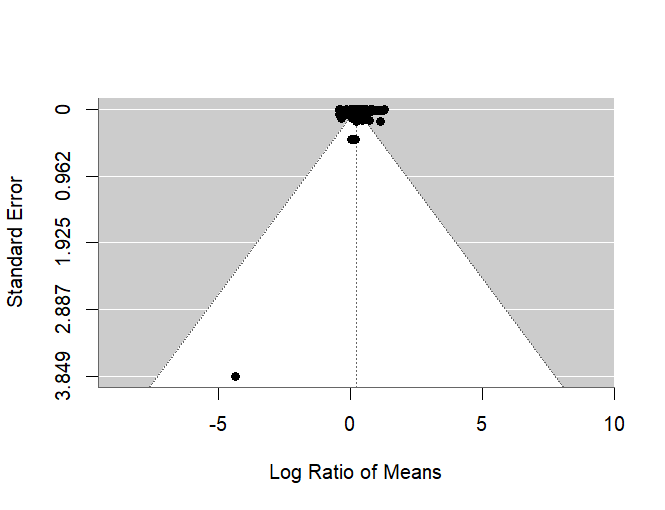

Supplement: Supplementary file 2 [file DataSheet1.zip › Funnel chart/Yield.png]
