# Supplementary material for: Multi-environment meta-analysis reveals the mechanism of action of potassium-solubilizing microorganisms on crop yields
Source: Front Plant Sci. 2025 Nov 3;16:1659478. doi: 10.3389/fpls.2025.1659478 (PMC12620454; doi:10.3389/fpls.2025.1659478)
Supplement: Supplementary file 3 [file DataSheet2.docx]

**Caption of figures**

[**Supplementary Figure.S1**](https://www.frontiersin.org/journals/plant-science/articles/10.3389/fpls.2025.1637092/full#hsm) Graphical abstract.

**Supplementary Figure.S2** Effect values of KSM inoculation on soil nutrients, soil pH, oxidation-related enzyme activities and metabolism-related enzyme activities (A); soil available potassium content and soil pH (B); superoxide dismutase, catalase and peroxidase (C).

**Supplementary Figure.S3** Effect values of growth indexes of inoculated KSM (A), plant height(B).


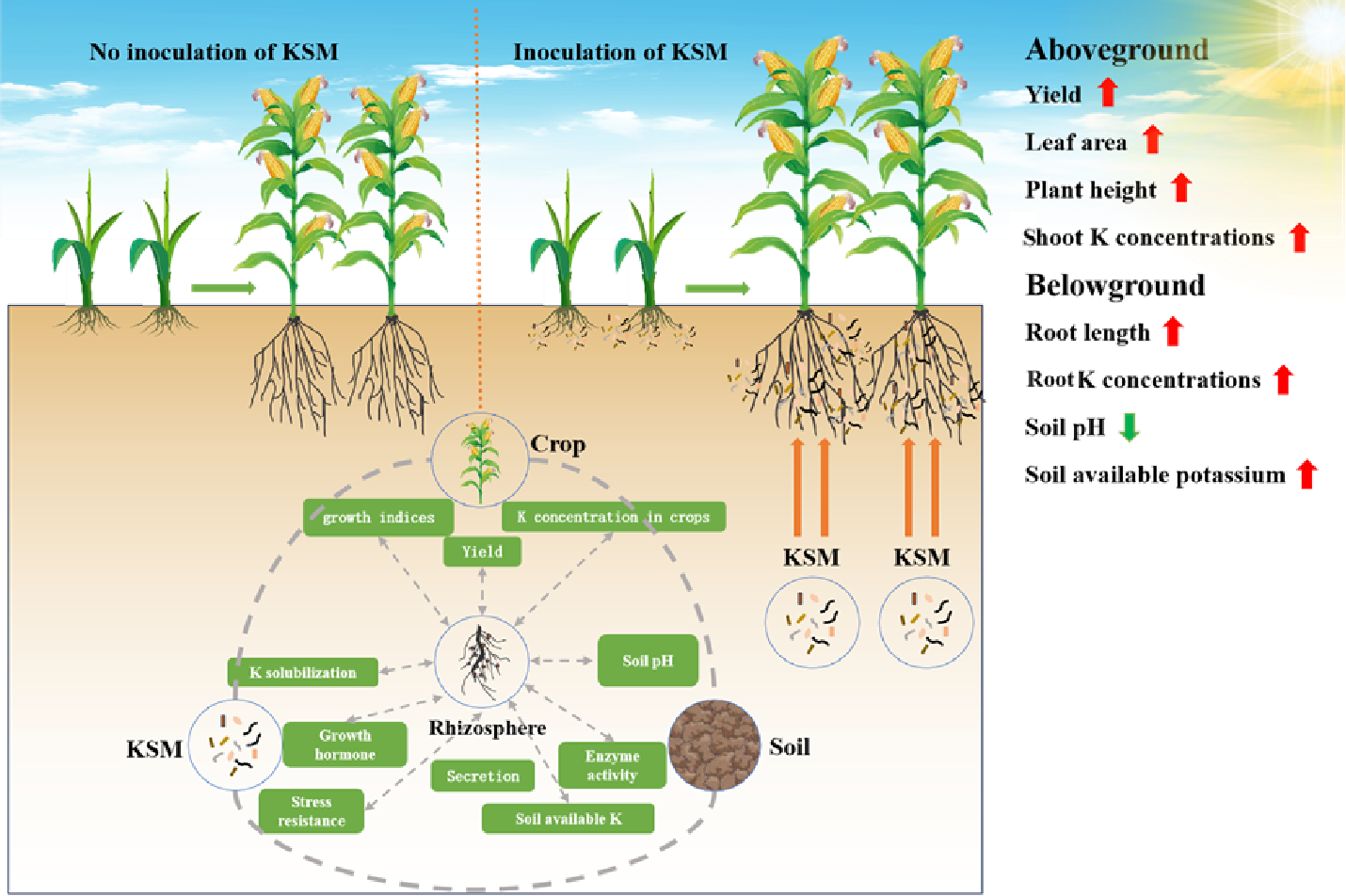


Supplementary Figure.S1


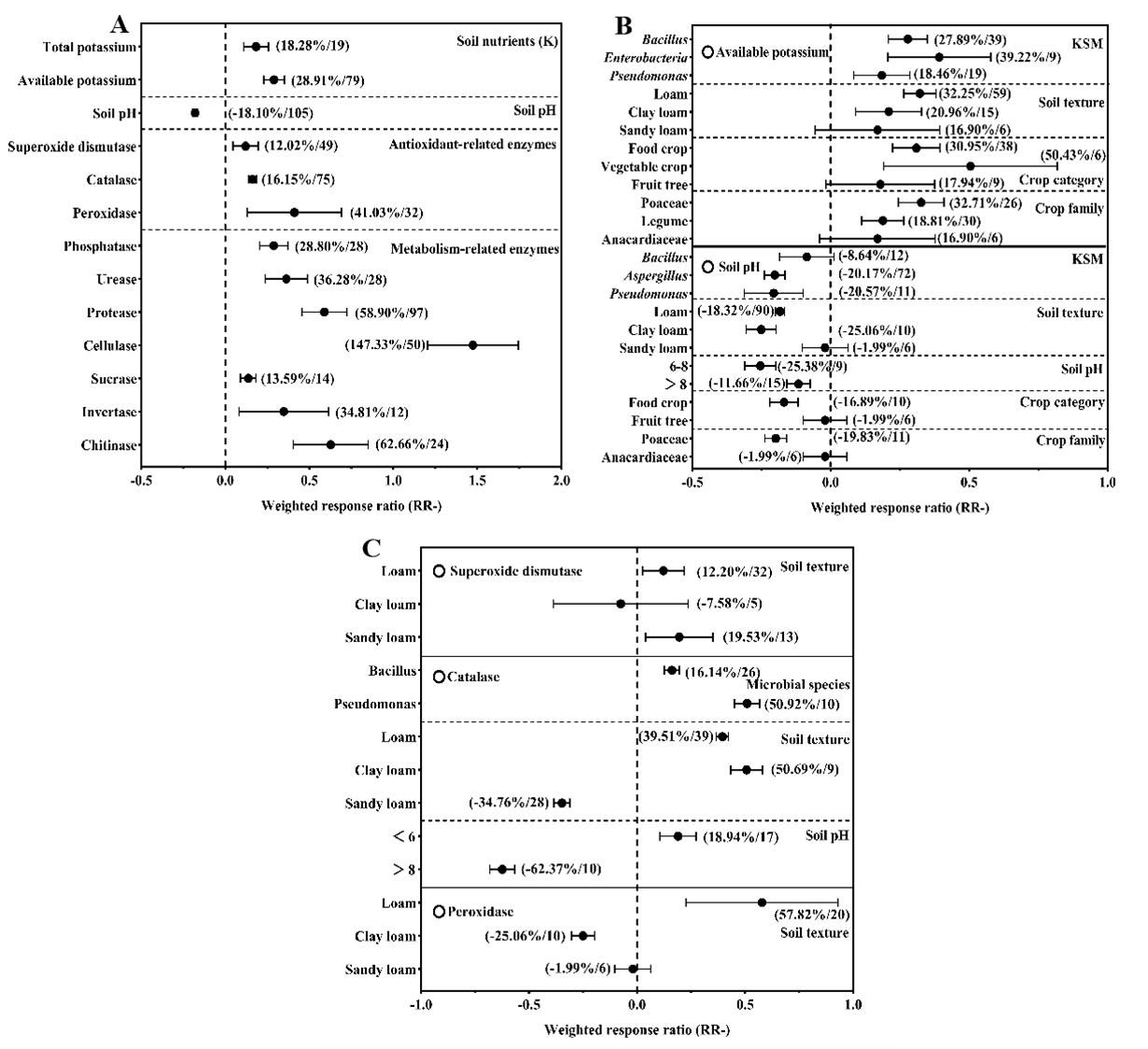


Supplementary Figure.S2

**
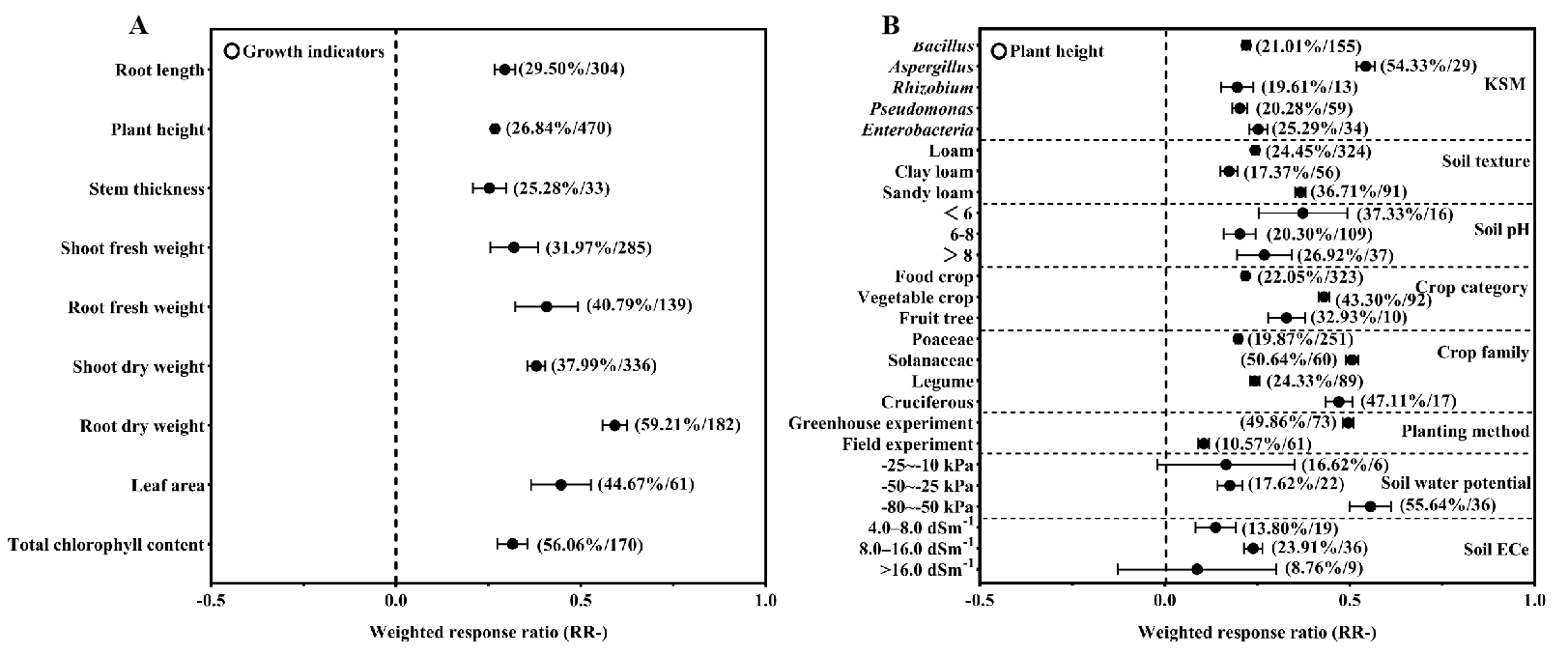
**

Supplementary Figure.S3
